# Application of Digital Tools in the Care of Patients With Diabetes: Scoping Review

**DOI:** 10.2196/72167

**Published:** 2025-08-19

**Authors:** Yan Yan, Na Li, Chun Tian

**Affiliations:** 1 School of Nursing, Shanxi Medical University, Taiyuan, China; 2 Department of Stomatology, First Hospital of Shanxi Medical University, No. 85, Jiefang South Road, Yingze District, Shanxi Province, Taiyuan, 030001, China, 86 1-383-461-1446

**Keywords:** digital technology, diabetes mellitus, nursing, scoping review, PRISMA, mobile phone

## Abstract

**Background:**

The global rise in the prevalence of diabetes significantly impacts the quality of life of both patients and their families. Despite advances in diabetes care, numerous challenges remain in its management. In recent years, digital tools have been increasingly integrated into diabetes care, demonstrating some positive outcomes. However, the long-term effectiveness and associated challenges of these tools in diabetes management remain areas for future research.

**Objective:**

This study aims to assess the types and current usage of digital tools in diabetes management, analyze their benefits and limitations, and provide recommendations for optimizing their future application in diabetes care.

**Methods:**

This scoping review followed the 5-stage framework proposed by Arksey and O’Malley. A comprehensive literature search was conducted across 9 electronic databases (CNKI, Wanfang Database, VIP, Sinomed, PubMed, Embase, Web of Science, CINAHL, and Cochrane Library) from their inception to July 31, 2024. Study selection was independently performed by 2 reviewers, descriptive analysis was conducted, and findings were presented narratively, key study characteristics—including first author, publication date, country or region, study type, sample size, type of digital tools, intervention methods, intervention duration, limitations, and outcome indicators—were independently extracted and cross-checked by 2 investigators.

**Results:**

A total of 6263 articles were initially retrieved. After deduplication and a 2-stage screening process (initial screening based on titles and abstracts followed by full-text assessment), 45 studies meeting the predefined inclusion criteria were ultimately included for analysis, originating from 7 countries. The included studies demonstrated marked heterogeneity in research designs: randomized controlled trials (RCTs; n=26, 57.8%), non-RCTs (n=15, 33.3%), quasi-RCTs (n=1, 2.2%), observational studies (n=1, 2.2%), mixed-methods studies (n=1, 2.2%), and qualitative studies (n=1, 2.2%). The digital tools primarily included mobile health apps, integrated management information platforms, Diabetes Online Community (DOC), specialized monitoring and analytics tools, blood glucose information management systems, and remote monitoring and follow-up systems, among others. These tools were applied across home, hospital, and community settings. Outcome measures were primarily focused on evaluating glycemic control efficacy (eg, fasting blood glucose, postprandial blood glucose, and glycated hemoglobin), blood lipid levels, BMI, self-management capacity, quality of life, patient satisfaction, and diabetes knowledge.

**Conclusions:**

This review highlights the diversity and potential value of digital tools in diabetes care, particularly in supporting patient self-management and extending care across multiple settings. From a nursing perspective, digital interventions offer opportunities for individualized care, patient engagement, and continuity of services. However, challenges such as technology acceptance remain. Future studies should address gaps related to long-term effectiveness, economic evaluation, and tool adaptation for aging populations, while incorporating interdisciplinary approaches and real-world evidence to inform sustainable digital health strategies.

## Introduction

The International Diabetes Federation (IDF) Diabetes Atlas [1] reports that approximately 537 million adults aged 20‐79 years were living with diabetes worldwide, a number projected to rise to 783 million by 2045, representing 10.5% and 12.2% of the global population in this age group, respectively. Notably, China accounts for over a quarter of the global diabetic population, ranking first worldwide. These alarming statistics reflect the rapid rise of diabetes and its severe impact on patients and their families.

Despite their demonstrated benefits, traditional approaches to diabetes care and self-management remain limited by persistent challenges, including long-term adherence to balanced diets and regular exercise, inaccuracies in medication timing and glucose monitoring, limited dissemination of care knowledge, and the heavy burden of daily self-management—all of which significantly constrain effective disease control [2-4].

To address these challenges, digital tools have gained substantial traction in diabetes care in recent years. Digital tools refer to software and devices that can collect, access, search, process, and use digital resources. Common digital tools include smartphone apps, information communication technologies, and wearable devices [5], offering novel approaches to overcoming traditional diabetes care limitations while improving patients’ quality of life and glycemic outcomes. One application of digital tools is in digital behavior change interventions (DBCIs), which refer to structured strategies using digital technologies—such as mobile apps, web platforms, and wearable devices (eg, activity trackers)—to modify specific health-related behaviors (eg, physical activity) [6]. In August 2021, the World Health Organization issued its inaugural Global Strategy on Digital Health (2020‐2025), outlining a vision, strategic objectives, and an action framework to advance digital health worldwide, emphasizing technology-enabled health care improvements [7]. Subsequently, the National Health Commission of China issued the National Nursing Development Plan (2021‐2025) in 2022, highlighting the critical role of nursing informatization and advocating for high-quality nursing development and systemic enhancements [8]. Nursing informatization is key to improving care quality and efficiency. It involves using modern information technologies, such as digital tools, artificial intelligence (AI), and mobile internet, to innovate care service models and management methods. Informatization can streamline nursing workflows and improve work efficiency. However, the broader implementation of digital tools faces multiple challenges. First, disparities in patients’ technological literacy and adaptability—particularly among older adults or technology-resistant groups—may reduce adherence due to complex interfaces and unintuitive functionalities [9]. Second, the digital divide restricts access to high-quality digital care resources in underserved regions, notably in developing countries and remote areas, thereby limiting tool adoption and effectiveness [10,11]. Furthermore, while AI integration enables personalized interventions and precision health management, it also introduces emerging challenges related to algorithmic transparency, data privacy protection, and ethical concerns, necessitating careful consideration in both technological development and policy formulation [12,13].

Given this context, this study adopts a scoping review methodology, which is well suited for mapping the diversity of emerging digital tools and their multidimensional applications, rather than focusing solely on efficacy outcomes. While most existing reviews target single categories of digital tools, our work addresses a critical literature gap by synthesizing multinational, cross-category evidence [14-18]. We systematically examine the types and current uses of digital tools in diabetes care, highlight their benefits in improving glycemic control, self-management, and care quality, and critically assess key implementation challenges—including technology acceptance, digital inequity, data security, and privacy concerns.

Notably, previous studies have primarily emphasized short-term outcomes. In contrast, this study introduces a novel perspective on digital chronic disease management by proposing the potential of digital interventions to delay diabetes-related complications—such as retinopathy and nephropathy—and by evaluating their economic feasibility, thereby broadening this study’s scope. Our findings offer both a theoretical foundation and practical insights to support the wider implementation of digital therapeutics in diabetes management.

## Methods

### Overview

This scoping review adopts the 5-step framework proposed by Arksey and O’Malley [19] and is reported according to the PRISMA-ScR (Preferred Reporting Items for Systematic Reviews and Meta-Analyses extension for Scoping Reviews) guidelines (see Checklist 1). During the development of the search strategy, we drew upon our in-depth understanding of the diabetes care field and preliminary investigations into research on digital tools. Specifically, we first deconstructed the research question using the PICOS framework (Participants: individuals with diabetes; Interventions: digital tools; Comparisons: traditional care models; Outcomes: glycemic control, quality of life, etc; Study design: randomized controlled trials, observational studies, etc). This framework provided a clear structure for subsequent keyword selection and strategy refinement.

We identified high-frequency and critical terms by reviewing seminal literature and review articles in the field. Initial keywords such as “diabetes,” “digital,” and “nursing” were extracted. To enhance comprehensiveness and precision, we expanded synonyms and optimized Boolean operators. For example, “Diabetes” was combined with synonyms like “glucose” and “blood sugar.” Boolean logic (AND/OR/NOT) was applied to refine the search strategy.

To verify the robustness of our approach, post hoc, we consulted separately with a medical librarian and an evidence-based nursing information specialist from Shanxi Medical University after submission. Their professional feedback confirmed that our keyword selections and overall strategy were methodologically appropriate and aligned with best practices for cross-language literature reviews involving both English and Chinese databases.

### Define Research Questions

The specific research questions proposed in this study are (1) What digital tools are currently used in diabetes care?,(2) How are digital tools applied in diabetes care?, and (3) What are the effects of using digital tools in diabetes care?

### Identify Relevant Studies

A literature search was conducted across 4 Chinese databases (CNKI, Wanfang Database, VIP, and Sinomed) and 5 English-language databases (PubMed, Web of Science, The Cochrane Library, CINAHL, and Embase), with the search period from database inception until July 31, 2024. In the English databases, a combination of subject terms and free terms was used for the search. For example, the search strategy for PubMed was: ((diabetes [Title/Abstract] OR blood sugar[Title/Abstract] OR glucose[Title/Abstract]) AND (digital[Title/Abstract] OR electronic[Title/Abstract] OR information[Title/Abstract] OR software[Title/Abstract] OR online[Title/Abstract])) AND (nursing[Title/Abstract] OR management[Title/Abstract]). The full search strategy can be found in Multimedia Appendix 1.

### Study Selection

The inclusion criteria (see Textbox 1) for the literature were determined based on the PCC (Participants, Concept, and Context) framework from the Joanna Briggs Institute [20].

Textbox 1. **Inclusion and exclusion criteria.**

Inclusion criteria:

- The study population consists of patients with diabetes.
- The concept involves providing digital tool interventions for patients with diabetes.
- The context includes patients receiving digital tool-based care in hospitals, communities, or at home.
- The study type is primarily experimental, observational, or another original research.

Exclusion criteria:

- Non-Chinese or non-English language literature.
- Full text not available.
- Incomplete or irrelevant data.
- Duplicate studies.
- Literature types such as conference abstracts or reviews.
- Guidelines, expert consensus, etc.

All retrieved citations were initially imported into EndNote software (Clarivate) for deduplication. Subsequently, 2 researchers (YY and NL), both equipped with knowledge of evidence-based nursing, independently screened the titles and abstracts in accordance with the inclusion and exclusion criteria. Meanwhile, the reference lists of the included literature were screened to identify additional eligible studies. Given that this was a scoping and not a systematic review of the literature, we did not conduct a formal quality appraisal of included studies using standardized tools. However, to ensure the reliability and interpretability of synthesized findings, we applied minimum methodological thresholds during the full-text screening phase. Specifically, studies were excluded if they exhibited major methodological deficiencies such as: (1) studies with extremely small sample sizes but no rationale (eg, fewer than 10 participants), (2) randomized controlled trials (RCTs) lacking sample size calculation or baseline comparability, (3) studies with vague or poorly described study designs (eg, unclear intervention processes or outcome definitions), (4) near-duplicate studies with substantial overlap in data or findings.

In addition, we found that the core concepts and topics presented in the excluded studies were already well-represented in the included literature, ensuring comprehensive thematic coverage despite the exclusion. Any discrepancies during screening were resolved through iterative discussions between reviewers or arbitration by a third researcher.

### Data Charting

During the data extraction phase, 2 reviewers (YY and NL) independently extracted the required information from the included studies using a predesigned data extraction form and cross-checked each other’s results in parallel. The extracted information included: the first author, publication date, country or region, study type, sample size, type of digital tools, intervention methods, intervention duration, limitations, and outcome indicators.

### Data Synthesis

Based on a systematic analysis of the included studies, we categorized digital tools into 6 types by considering their core functionalities (such as data collection, decision support, and patient education), typical application scenarios in diabetes care (eg, home monitoring and hospital-based management), and alignment with practical nursing workflows and patient needs.

## Results

### Organize, Summarize, and Report Findings

**Figure 1.**
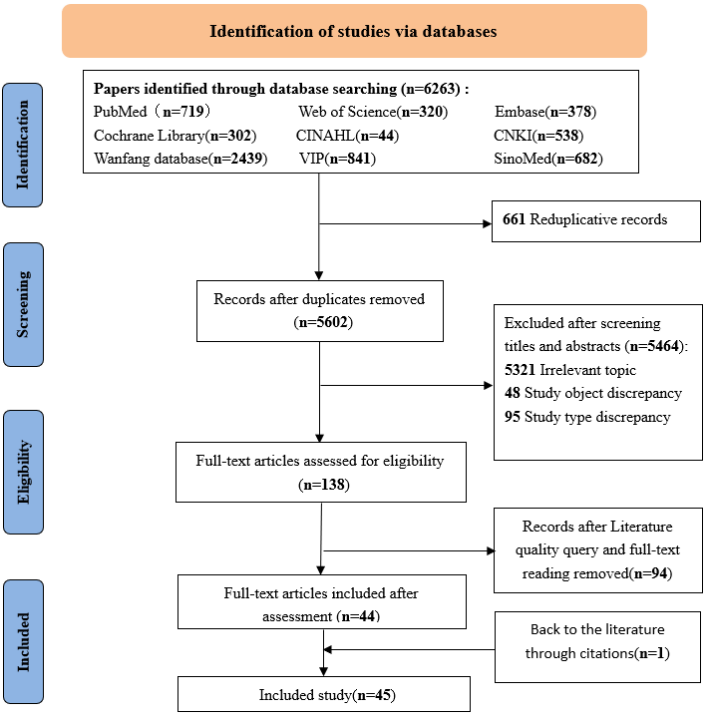
The PRISMA-ScR (Preferred Reporting Items for Systematic Reviews and Meta-Analyses extension for Scoping Reviews) flowchart illustrating the study selection process.

We initially identified 6263 records through database searching. After removing 661 duplicates, 5602 records remained. Based on the inclusion and exclusion criteria, 5464 records were excluded after screening titles and abstracts, including 5321 that were irrelevant to the topic, 48 with ineligible study populations, and 95 with ineligible study types. The remaining 138 articles underwent full-text review and quality assessment, resulting in the exclusion of 94 low-quality studies. An additional study was manually added during reference list screening. Ultimately, 45 studies were included in the review [21-65]. Figure 1 shows the PRISMA-ScR flow diagram, and the PRISMA-ScR checklist is provided in Checklist 1.

### Basic Characteristics of Included Studies

This review included a total of 45 studies, which were conducted in 7 countries: China (n=36, 80%), South Korea (n=3, 6.6%), the United States (n=2, 4.4%), Germany (n=1, 2.2%), Israel (n=1, 2.2%), Ireland (n=1, 2.2%), and India (n=1, 2.2%). The study types included RCTs (n=26, 57.8%), non-RCTs (n=15, 33.3%), quasi-randomized studies (n=2, 2.2%), observational studies (n=1, 2.2%), and mixed-methods studies (n=1, 2.2%). The sample sizes were mostly between 80 and 280 participants, and the study durations were typically 3 to 6 months. The study populations primarily consisted of patients with type 2 diabetes, with some studies focusing on older adults or on middle-aged and young patients; the populations also included discharged patients and community residents. Basic characteristics are provided in Multimedia Appendix 2.

### Use Scenarios, Distribution of Digital Tool Types, and Application Analysis in Diabetes Management

Digital tools were primarily used in home-based settings (27/45, 60%) [21-23,27,29,31,32,35,36,38-41,43,47,48,50-56,59,62,63], followed by hospital environments (8/45, 18%) [28,33,37,46,49,54,60,61]. Other usage contexts included community settings (2/45, 4%) [58,65], home and community settings (4/45, 9%) [24,25,30,57], home and hospital settings (3/45, 7%) [26,44,64], and all 3 settings (1/45, 2%) [42]. In descending order of frequency, these were home use, hospital use, home and community use, home and hospital use, community use, and use in all 3 settings.

The research findings indicate that the types of digital tools included in the studies show significant distribution differences. Among these, mobile health apps (21/45, 47%) [22-24,26,33,34,36,39-41,45,47,48,51,53,55,57-59,62,65] account for the highest proportion, followed by blood glucose information management systems (BGIMS; 10/45, 22%) [29-32,37,43,44,46,49,54], integrated information management platforms (7/45, 16%) [25,27,28,35,38,42,56], professional monitoring and analysis tools (3/45, 7%) [21,60,61], remote monitoring and follow-up systems (3/45, 7%) [50,52,64], and Diabetes Online Community (DOC) (1/45, 2%) [63]. A summary of the research findings on digital tools in the included literature is provided in Table 1.

**Table 1.** Summary of research findings on digital tools in the included literature.

Digital tool type	Functional features	Main research findings	Limitations
Mobile health apps	Real-time monitoring, data recording, and online consultation.	Enhances patients’ self-management capabilities [22,41,55,57] and improves glycemic control [22,34,40,41,45,47,51,53].	Low acceptance among older adults [23,26,33,34,53,57,65].
Integrated information management platform	Information integration, multiplatform design, and multidisciplinary clinical care.	Improves accessibility and efficiency of health care services [25,27,35,42,56].	Difficult to implement with high technical support requirements [25,42].
Diabetes Online Community	Provides emotional support and health education.	Enhances patients’ confidence in self-management [63].	Not reported.
Professional monitoring and analysis tools	Monitors physiological parameters; provides scientific evidence.	Optimizes treatment plans and offers precise data support [21,60,61].	Not reported.
Blood glucose information management system	Real-time blood glucose monitoring, cloud storage, and analysis.	Reduces nursing workload and improves treatment decision-making efficiency [29,32,37,43,44,46,49,54].	Not reported.
Electronic remote monitoring and follow-up systems	Remote health status monitoring with timely interventions.	Improves patients’ self-management capabilities and patient satisfaction [52,64].	Implementation is challenging initially and increases nursing workload [64].

Analysis of the included studies revealed that the use of digital tools was associated with significant improvements across multiple patient-related health indicators. Specifically, 33 studies (73%) reported a significant reduction in glycated hemoglobin (HbA_1c_) levels [21-25,28-32,34-36,38-45,47,49-53,56-58,62,64,65], 26 studies (58%) found improvements in fasting plasma glucose (FPG) [21,22,24,25,27-30,32,34-36,38-42,44,45,49,51-54,56,62], and 21 studies (47%) [21-23,25,27,28,30,32,34,38-42,44,45,49,51-53,56] showed reductions in 2-hour postprandial glucose (2hPG). In addition, 12 studies (27%) [21,25,30,34,36,39,42,45,49,51,56,57] reported lowered triglyceride levels, 10 studies (22%) [21,25,29,30,39,42,45,49,51,56] found reductions in total cholesterol, and 9 studies (20%) [21,22,25,34,36,42,57,60,62] observed improvements in BMI. Several studies also reported increases in diabetes-related knowledge [26,32,33,38,40,49,50,52,56], better self-management capabilities [22,25,27,30,41,55,57], and improved patient satisfaction [26,28,30,44,48,50,58]. Furthermore, 7 studies (16%) [30,31,37,40,44,50,54] reported fewer hypoglycemic episodes following digital intervention. It is worth noting that 9 studies (20%) [23,25,26,33,34,42,53,57,65] have pointed out the specific obstacles that older adults encounter when using digital tools, such as their physiological characteristics (eg, poor eyesight), limited digital literacy, and complicated user interfaces.

### Mobile Health (mHealth) Apps

A total of 21 studies (21/45, 47%) [22-24,26,33,34,36,39-41,45,47,48,51,53,55,57-59,62,65] focused on the use of mHealth apps in diabetes care. Among them, 14 studies [26,34,36,39-41,47,51,53,55,57,59,62,65] examined diabetes management apps, 1 study [22] evaluated a home care app, 2 [23,45] involved WeChat public accounts, 1 [33] assessed a subscription account, 1 [58] used SMS interventions, and 3 [22,24,48] explored and “Internet Plus” health management platforms. These tools offered a variety of features, including dietary logging (9/21, 43%) [22,24,26,34,39,41,51,53,57], blood glucose reminders (11/21, 52%) [22-24,40,45,51,53,55,57-59], medication reminders (8/21, 38%) [22,24,34,39,41,45,53,58], exercise planning (7/21, 33%) [22,24,36,41,51,53,57], personalized meal plans (3/21, 14%) [26,36,51], feedback on health indicators (12/21, 57%) [22-24,34,40,41,47,53,55,57-59], and insulin injection site rotation reminders (1/21, 5%) [22]. Some apps also supported doctor-patient communication (10/21, 48%) [22-24,39,41,45,48,51,53,57], knowledge dissemination (14/21, 67%) [22,24,33,34,36,39-41,45,47,51,53,57,59], and personalized feedback (4/21, 19%) [22,24,41,59].

Regarding dietary management, 4 studies [26,34,36,51] reported improvements in blood glucose and lipid levels after using mHealth-based dietary interventions. A total of 3 studies [40,48,55] found that mHealth tools helped improve glycemic control, enhance health knowledge, and promote self-management behaviors. In addition, 2 studies [33,45] highlighted the role of app-delivered educational content in strengthening self-management. Furthermore, 4 studies [22,39,50,51] suggested that mHealth tools could improve follow-up efficiency and support better glycemic outcomes.

Some studies focused on older adults with type 2 diabetes. For instance, Deng et al [23] evaluated a smartphone-based health management system for patients aged 60 years and older and observed improved glycemic control. Dr. Sunhee Park’s research team [65] developed the DiaNote app specifically for older South Korean patients with type 2 diabetes. The study reported a decrease in HbA_1c_ levels after using the app.

### Integrated Management Information Platform

A total of 7 studies (7/45, 16%) [25,27,28,35,38,42,56] examined the implementation of an integrated diabetes management information platform, which typically includes functional modules such as blood glucose monitoring, dietary recording, exercise management, and medication reminders. Among these, 6 studies (6/7, 86%) [25,27,28,35,38,42] explicitly highlighted the establishment of a multidisciplinary team to deliver comprehensive management for patients with diabetes.

Furthermore, 3 studies (3/7, 43%) [25,35,42] reported achieving seamless one-stop management through the development of 3 portals: health care provider, patient, and community hospital portals, which ensure the integration of information across hospital, community, and home settings.

### Diabetes Online Community (DOC)

Among the 45 included studies, only 1 (2%) examined the role of the DOC in supporting older adults with diabetes. In this qualitative study, Litchman et al [63] interviewed 20 older patients with diabetes to explore their motivations for engaging in DOC and their interactions with health care providers. The study identified DOC as a form of peer health support that provided access to diabetes-related information, emotional support, and a sense of community belonging. Participants reported that DOC helped enhance their self-management skills through peer interaction and experience sharing. Notably, many used DOC as a supplementary resource between medical appointments, helping to maintain consistent self-care during periods without direct provider contact. Some participants noted that the empathy and shared understanding found in DOC often exceeded the support they received from health care professionals or family members. However, concerns were also expressed about the potential emotional impact of negative posts or comments within the community.

### Specialized Monitoring and Analysis Tools

A total of 3 studies (3/45, 7%) [21,60,61] reported the application of professional monitoring and analysis tools in diabetes management, primarily functioning in 2 domains: quantifying behavioral interventions and enhancing treatment adherence. A 3-month self-controlled study [21] observed downward trends in patients’ body weight, BMI, waist circumference, blood glucose levels (both fasting and postprandial), HbA_1c_, and lipid profiles (including total cholesterol, triglycerides, and low-density lipoprotein) following the use of a wearable energy monitoring device (which is typically worn around the waist to monitor the user’s physical activity) and its associated system software. In addition, improvements were noted in effective physical activity duration, dietary control, and energy balance.

In addition, 2 studies from Korea [60,61] evaluated the use of the OTDMS (Online Tracking and Data Management System), which tracks and monitors blood glucose levels by connecting glucose meters and computers, transforming recorded data into charts and statistics. Although OTDMS did not demonstrate significant differences in diabetes knowledge, patient satisfaction, or HbA_1c_ levels compared to control groups, it showed positive effects on increasing the frequency of weekly glucose monitoring and improving adherence to medical instructions.

### Remote Monitoring and Follow-Up System

Among the reviewed literature, 3 studies (3/45, 7%) [50,52,64] have demonstrated the significant effectiveness of remote monitoring and follow-up systems. A study [52] found that patients using an electronic information follow-up system exhibited markedly improved insulin knowledge, biochemical control, and treatment adherence compared to those receiving conventional postdischarge follow-up. Another study [50] indicated that patients managed through WeChat in conjunction with the “Micro Sugar” app experienced greater reductions in HbA_1c_ levels, weight loss, increased frequency of blood glucose monitoring, and higher satisfaction levels compared to those receiving traditional telephone follow-up. A third study [64] revealed that remote monitoring via MyMedic hubs installed in patients’ homes, coupled with telephone support from clinical nurse specialists for insulin dose adjustments, resulted in significant declines in HbA_1c_ levels, elevated scores on the Diabetes Empowerment Scale (DES), reduced scores on the Diabetes Distress Scale (DDS), and patient satisfaction ratings exceeding 4 out of 5. However, this approach also resulted in an increased workload for health care professionals.

### Blood Glucose Information Management System

A total of 10 studies (10/45, 22%) [29-32,37,43,44,46,49,54] reported on the application of BGIMS in diabetes care. These systems typically comprise smart glucose meters (eg, GLUPAD) connected to computer terminals, facilitating automatic data collection and real-time synchronization of blood glucose levels. Several studies [29-32,37,43,44,46,49,54] observed a downward trend in HbA_1c_, FPG, and 2hPG, following the implementation of BGIMS. A study [37] noted that the system supported daily glucose monitoring and management, reduced the workload of health care staff, and enhanced overall management efficiency.

A total of 2 studies [31,32] reported that the use of BGIMS increased the frequency of self-monitoring of blood glucose (SMBG), enabling patients to detect abnormal glucose fluctuations and adjust their management accordingly. A study by Zhao et al [54] further explored the potential of BGIMS to improve provider knowledge, standardize diabetes health education, enhance patient adherence, and alleviate financial burdens. The authors suggested expanding the application of BGIMS to additional clinical departments.

## Discussion

### Principal Findings

This scoping review systematically explored the application of digital tools across inpatient, community, and home settings for patients with diabetes. Our study revealed that various digital interventions—including mHealth apps, integrated management information platforms, remote monitoring systems, the DOC, and specialized monitoring tools—can significantly improve patients’ blood glucose control, self-management capabilities, and quality of life. The review emphasizes a nursing perspective by integrating digital tools through a “hospital–community–home” continuum of care, thereby addressing previous shortcomings of predominantly medically oriented approaches that lacked practical nursing guidance.

### Comparison With the Existing Literature

Based on a systematic analysis of the included studies, we categorized digital tools into 6 types by considering their core functionalities (such as data collection, decision support, and patient education), typical application scenarios in diabetes care (eg, home monitoring and hospital-based management), and alignment with practical nursing workflows and patient needs. During the classification process, we observed that many tools inevitably featured overlapping functions. This is largely due to their integrated design, which aims to address the complex and multifaceted requirements of diabetes care. For example, mHealth apps often combine data tracking, educational content, and even preliminary decision-support features. Similarly, integrated management platforms may include data processing capabilities along with patient education modules. The purpose of our classification was to highlight the primary role and focus of each tool type within practical care environments, rather than to imply mutual exclusivity.

Existing reviews have often focused on a single type of tool (such as mHealth apps) or on specific populations (eg, older adult patients) [66-69]. In contrast, this study systematically covers 6 categories of digital tools and classifies them from the perspective of nursing services, emphasizing the selection of appropriate tools within actual nursing workflows. Unlike most reviews that focus solely on blood glucose indicators, this study also examines the effects of digital tools on comprehensive outcomes such as patient satisfaction, self-efficacy, and quality of life, thereby reflecting a multidimensional, patient-centered approach from a nursing perspective.

### Advantages of Digital Tools in Diabetes Management

#### Enhancing Self-Management Capabilities in Patients With Diabetes

mHealth apps and integrated management information platforms provide real-time monitoring, data recording, and online consultation, offering personalized management suggestions. For example, Dai et al [22] developed an internet-based diabetes home care app for managing health in the experimental group. The app includes 3 interfaces: for health care providers, supervisors, and patients, offering interaction, responses to questions, as well as advice on medication, diet, exercise, and emergency situations. Another research study developed an app-based “hospital–community–home” care plan for patients with type 2 diabetes, aiming to enable continuous and personalized management. The approach was found to support resource integration and complementary advantages [70].

In today’s health care environment, the adoption of multidisciplinary collaboration to manage diabetes has also shown significant benefits [28,42]. In the future, diabetes management could involve building multidisciplinary teams, integrating medical information systems, and using mHealth apps and cloud platforms for data management. This would enable continuous care, promote patient engagement, and support ongoing research and innovation, creating an efficient, coordinated, and patient-centered management system that enhances blood glucose control and self-management abilities, while improving health outcomes and quality of life.

In addition, online interactions in diabetes communities positively impact the quality of self-care, an aspect that hospital treatments alone cannot achieve. Such online interactions indirectly influence diabetes self-management quality through self-efficacy [71]. Therefore, in clinical diabetes care, it is important to encourage patients to interact with each other to enhance the effectiveness of optimal diabetes control.

#### Improving Accessibility and Convenience of Health Care Services

Smartphone health apps make diabetes management more effective and rational, improving patients’ self-management abilities and blood glucose control [39,41]. These findings are consistent with those reported by Agnes et al [66]. Integrated management information platforms and electronic remote monitoring systems enhance health care service accessibility by integrating medical resources and enabling remote monitoring. Patients can transmit their data to health care providers at home via electronic devices, enabling providers to offer remote support and advice [64].

Given these advantages, future diabetes management should make use of these technologies, enabling real-time monitoring of blood glucose and metabolic indicators, while providing timely, personalized disease guidance. Products should be designed to accommodate the physical and mental characteristics of various age groups, thus achieving more effective diabetes management.

#### Optimizing Treatment Plans

With advancements in technology, diabetes monitoring tools have continually evolved, from traditional blood glucose meters to continuous glucose monitoring (CGM) systems, smart insulin pumps, and wearable devices. Their application makes diabetes management more precise and efficient. Specialized monitoring tools play a crucial role in diabetes care by collecting and analyzing patients’ blood glucose and other physiological parameters in real-time, providing physicians with scientific data to optimize treatment plans. These tools not only monitor blood glucose levels but also correlate with other health indicators such as blood pressure and weight, offering a comprehensive health management solution for patients.

In the future, we can further explore and use these professional monitoring tools, combining them with AI technology to achieve more precise and comprehensive diabetes management.

### Challenges in the Application of Digital Tools in Diabetes Care

Although digital tools have demonstrated enormous potential in diabetes nursing, their practical application still faces multiple challenges.

First, patients’ acceptance of technology poses a significant barrier, particularly among older adults. Numerous studies have shown that older patients often exhibit reduced adherence due to issues such as small fonts, complex interfaces, or the need for manual data entry, which significantly limits their effective management [23,25,26].

Second, the ease of use and reliability of technology directly affect the intervention outcomes. The study by Patail et al [68] indicates that although mobile apps can effectively enhance self-management confidence among patients with diabetes, technical obstacles—such as compatibility issues, operational complexity, and a lack of personalized design—often hinder long-term use.

Third, concerns over data security and privacy protection are becoming increasingly prominent. Most of the included studies did not elaborate on encryption measures and access control mechanisms, while the research by Liu et al [72] found that nursing information security faces various threats including hacker attacks, virus infections, and technical flaws. This necessitates the implementation of robust policies and technologies to safeguard patient information.

Finally, the promotion of digital tools is constrained by the IT literacy of health care professionals. The development of smart nursing requires that nursing staff possess a certain level of information technology and AI knowledge. However, the overall IT proficiency among nursing personnel is currently relatively weak, making the enhancement of relevant training and education another challenging issue [73].

### Implications for Future Research

Based on the limitations of current studies and the practical demand for digital transformation in diabetes management, future research needs to establish a multidimensional, hierarchical evidence production system. Breakthroughs should be pursued in the following 4 directions:

#### Optimization of Research Design

For an extended follow-up and expanded study scale, it is recommended to prioritize multicenter RCTs with follow-up periods of at least 12 months to systematically assess the long-term effects of digital tools on blood glucose control, sustained adherence, behavior change, quality of life, and patient satisfaction. Simultaneous cost-effectiveness analyses should be conducted to evaluate the economic feasibility and resource allocation efficiency of these tools in different health care settings.

#### Development of a Personalized Intervention System

Adaptive technological solutions are required for older adult patients, individuals with limited digital literacy, and people with disabilities; hence, digital tools should be designed with age-friendly interfaces, voice interaction capabilities, and fault-tolerant mechanisms. Interface optimization can be guided by cognitive load theory—for example, by using progressive information presentation or setting up automatic correction functions for operational errors.

For the expansion of population coverage, there is an urgent need to conduct targeted studies on type 1 diabetes, gestational diabetes, and adolescent patients. Customized intervention modules that correspond to different physiological characteristics and behavior patterns should be developed. A mixed-methods research design is recommended to integrate quantitative efficacy evaluations with qualitative demand analyses. A study by Kaur et al [74] indicates that buckwheat extracts can inhibit sucrase activity and lower blood glucose, showing potential for diabetes management. Future research could explore using digital tools to promote dietary therapies, such as developing apps with buckwheat-rich recipes, to offer patients personalized dietary advice.

#### Technological Innovation and Application Enhancement

Advancing intelligent technology integration helps explore the application of AI in risk prediction, develop digital twin systems for simulating personalized interventions, and regularly updating intervention strategies based on data analysis.

Innovating health education formats, like technologies such as virtual reality (VR), can broaden the formats of health education for patients with diabetes. Hospitals may transform standardized in-house video resources into a shared learning platform for both patients and caregivers, thereby enhancing patient access to health knowledge and self-management capabilities.

Diabetic retinopathy (DR), a microvascular complication of diabetes mellitus (DM), is a leading cause of vision loss worldwide [75]. Diabetes also increases the risk of end-stage kidney disease [76]. These complications can lead to significant medical expenses and a deterioration in the quality of life. Our study shows that digital tools have demonstrated potential in improving short-term glycemic control and self-management; however, there remains a significant lack of research evaluating their long-term clinical outcomes and economic implications, particularly in preventing or delaying diabetes-related complications. Among the 45 included studies, none explicitly assessed the impact of digital interventions on the progression of complications such as diabetic retinopathy or nephropathy. Likewise, formal economic evaluations, including cost-effectiveness and budget impact analyses, were absent. This evidence gap limits understanding of the true value and scalability of digital therapeutics in real-world settings. Quantifying the input-output ratio of digital tools in reducing hospitalization rates and the incidence of complications will provide evidence-based support for health policy formulation. Future research should adopt complication-oriented strategies that use digital tools to incorporate preventive features—such as continuous monitoring, automated alerts, and personalized education—for early detection and intervention. Embedding reminders for routine fundus and kidney function examinations may improve adherence to surveillance protocols, mitigate complication risks, and optimize long-term health outcomes and resource use.

Within health care innovation, nurses must continuously innovate to address older adults’ physiological and psychological characteristics. Designing simple, engaging digital disease management approaches—under nurses’ leadership in ideation, design, implementation, and evaluation—can support patients in gradually improving self-care behaviors and strengthen nurses’ professional identity. Accordingly, by integrating traditional practices with modern technology—using the traditional Chinese medicine–derived finger cun [77] and 5-finger positioning methods [78]—we can develop straightforward and practical insulin injection assistance tools. Based on this, we can further explore the application of digital tools in diabetes management by developing simple and practical injection-assistance technologies that meet real-world nursing needs. Research by Huang [77] shows that the 5-finger positioning method is a convenient and easy-to-remember technique for diabetic patients requiring long-term insulin injections. It also aligns with the 4 injection sites specified in the 2016 edition of the “China Diabetes Medication Injection Technology Guidelines” [79]. This method is easy for patients to master, reduces the incidence of subcutaneous nodules, and improves adherence to and comfort with insulin injections. In the future, relevant applications can be developed to integrate the 5-finger positioning method into digital education content through animations and videos. These applications can also provide real-time guidance and feedback to help patients perform insulin injections more accurately and further enhance their self-management abilities.

We propose designing a simulated dial embedded within a digital tool interface to guide patients in rotating their insulin injection sites using the finger curl and the 5-finger positioning methods. The circular disk visually segments each injection zone, allowing users to rotate and point to the next site in sequence with ease. This design may reduce subcutaneous tissue damage and lipohypertrophy resulting from repeated injections at the same location. Furthermore, the intuitive visual aid can enhance older adults’ confidence in self-management, ultimately improving adherence and clinical outcomes. Future research will explore digital integration—such as providing real-time guidance and feedback via a mobile app or synchronizing with wearable devices to monitor injection behavior and outcomes—to deliver more comprehensive, personalized diabetes management support for older adults.

#### Improvement of Implementation Strategies

Establish a collaborative innovation mechanism by developing interdisciplinary research alliances (including fields such as clinical medicine, nursing, computer science, and health economics) to jointly formulate clinical translation pathways for digital therapeutics. An iterative “design–implementation–evaluation” model is recommended to ensure that the development of tools dynamically aligns with clinical needs.

Enhance supportive policy environments by promoting the establishment of a digital health technology certification system, develop data interoperability standards, and include digital tools in medical insurance reimbursement catalogs. In primary health care settings, complementary initiatives should be implemented to enhance health care professionals’ digital skills and patients’ technology acceptance.

Ethics and privacy protection can be strengthened by advanced encryption techniques, such as homomorphic encryption, and can be used to secure data transmission, while blockchain technology can be used to establish traceable data usage audit mechanisms.

### Limitations

First, there is a notable geographic imbalance in the literature concerning digital tools for diabetes care. The included studies exhibit a marked concentration in specific regions, particularly with China contributing a substantial proportion of the RCTs. This imbalance restricts the cross-cultural applicability of the findings and may not fully reflect the effectiveness and challenges of digital tools in other parts of the world. Future research should aim to conduct studies in a broader range of geographic locations to improve external validity of the results.

Second, many included studies had relatively short follow-up durations, predominantly ranging from 3 to 6 months, and exhibited low patient dependency, which hampers the assessment of the long-term benefits of digital interventions. Most studies enrolled between 80 to 280 participants, highlighting a significant lack of large-scale, multicenter effectiveness research.

Third, the current evidence base reveals considerable knowledge gaps. Economic evaluation data are limited, as most studies failed to report cost-effectiveness indicators, thereby restricting their applicability in health care decision-making across various resource allocation scenarios. The diversity of study populations is also constrained, with the majority of participants being individuals with type 2 diabetes. There is a relative scarcity of research addressing type 1 diabetes or specialized populations such as children and pregnant women with gestational diabetes.

Finally, we acknowledge that the consultation with information professionals was conducted retrospectively, rather than during the initial development of the search terms and strategy. This constitutes a methodological limitation of this review. In future studies, we will proactively engage information specialists at the early stages of the search strategy design, as their expertise can enhance the systematicity and authority of the literature search process. Incorporating such professional input will help us optimize our workflow and improve the methodological rigor and reproducibility of our research.

These limitations restrict the generalizability of the findings. Future research should aim to build a more comprehensive evidence ecosystem for digital diabetes management by establishing collaborative networks grounded in big data analytics, implementing follow-up periods of at least 12 months, incorporating health technology assessment frameworks, and conducting adaptive studies in special populations.

### Conclusions

This scoping review systematically maps the recent landscape of digital tools in diabetes care. The findings demonstrate that various digital tools have positively impacted glycemic control, self-management skills, health literacy, and quality of life among individuals with diabetes. These tools not only enhance the accessibility and efficiency of care by bridging hospital, community, and home settings but also provide personalized support for patient-centered disease management.

Notably, our study offers a novel perspective by reviewing 6 categories of digital tools within the hospital–community–home continuum—an area that has not been comprehensively addressed in previous literature. This approach fosters a more integrated understanding of how digital tools can be embedded across care settings to enhance diabetes management.

Future research should investigate the application of digital tools in other chronic conditions, such as cardiovascular disease and obesity. Furthermore, integrating emerging technologies—such as AI and blockchain—into digital health tools may further strengthen care delivery. There is also an urgent need to develop robust, evidence-based guidelines to support the implementation of digital tools in clinical nursing practice.

## Supplementary material

10.2196/72167 Multimedia Appendix 1 Search strategy.

10.2196/72167 Multimedia Appendix 2 The basic characteristics of the included literature.

10.2196/72167 Checklist 1 PRISMA-ScR (Preferred Reporting Items for Systematic reviews and Meta-Analyses extension for Scoping Reviews) checklist.

## Abbreviations

2hPG: 2-hour postprandial glucose
AI: artificial intelligence
BGIMS: blood glucose information management system
CGM: continuous glucose monitoring
DBCIs: digital behavior change interventions
DDS: Diabetes Distress Scale
DES: Diabetes Empowerment Scale
DM: diabetes mellitus
DOC: Diabetes Online Community
DR: diabetic retinopathy
FPG: fasting plasma glucose
GP: general practitioner
HbA_1c_: glycated hemoglobin
IDF: International Diabetes Federation
mHealth: mobile health
OTDMS: Online Tracking and Data Management System
PRISMA-ScR: Preferred Reporting Items for Systematic Reviews and Meta-Analyses extension for Scoping Reviews
RCT: randomized controlled trial
SMBG: self-monitoring of blood glucose
