# Supplementary material for: Application of Digital Tools in the Care of Patients With Diabetes: Scoping Review
Source: J Med Internet Res. 2025 Aug 19;27:e72167. doi: 10.2196/72167 (PMC12364422; doi:10.2196/72167)
Supplement: Multimedia Appendix 1 [file jmir-v27-e72167-s001.doc]

**Appendix 2. Search strategy.**

| **Database** | **Search Strategy** |
| --- | --- |
| PubMed | #1 "diabetes" OR "blood sugar" OR "glucose".ti  #2 "digital"OR "electronic" OR "information" OR "software" OR "online".ti  #3 "nursing" OR "management".ti.ab  #4 #1AND #2AND #3 |
| Embase | #1 'diabetes' OR 'blood sugar' OR 'glucose':ti  #2 'digital' OR 'electronic' OR 'information' OR 'software' OR 'online':ti  #3 'nursing' OR 'management':ti  #4 #1 AND #2 AND #3 |
| Cochrane Library | #1 "diabetes" OR "blood sugar" OR "glucose": ti  #2 "digital" OR "electronic" OR "information" OR "software" OR "online": ti  #3 "nursing" OR "management": ti ab kw  #4 #1 AND #2 AND #3 |
| Web of Science | #1 (ti=("diabetes" OR "blood sugar" OR "glucose")) OR ab=("diabetes" OR "blood sugar" OR "glucose")  #2 (ti=("digital" OR "electronic" OR "information" OR "software" OR "online")) OR ab=("digital" OR "electronic" OR "information" OR "software" OR "online")  #3 (ti=("nursing" OR "management")) OR ab=("nursing" OR "management")  #4 #1 AND #2 AND #3 |
| CINAHL | #1 ti=( diabetes OR blood sugar OR glucose ) AND ti= ( digital OR electronic OR information OR software OR online ) AND ti (nursing OR management ) |
| Wan fang | #1 主题:("糖尿病") OR 主题:("血糖")  #2主题:("数字") OR 主题:("电子") OR 主题:("信息") OR 主题:("软件") OR 主题:("在线")  #3 #1AND #2 |
| VIP | #1 题名="糖尿病 " OR 题名="血糖"OR题名="血葡糖糖"  #2 题名="数码" OR 题名="数字"OR题名="电子" OR 题名="软件"OR题名="在线"  #3 #1 AND # 2 |
| CBMdisc | #1 标题:("糖尿病") OR 标题:("血糖") OR 标题:("血葡萄糖")  #2标题:("数码") OR 标题("数字") OR 标题:("电子") OR 标题:("信息") OR 标题:("软件") OR 标题:("在线")  #3 #1AND #2 |

**Appendix 2** (Continued)

| **Database** | **Search Strategy** |
| --- | --- |
| CNKI | #1 主题="糖尿病" OR 主题="血糖"  #2 全文="数字" OR 全文="电子" OR 全文="信息" OR 全文="软件" OR 全文="在线"  #3 #1 AND #2 |

ti: title; ab: abstract; kw: keyword; jt: journal name; it: publication type ; nc: conference name
